# Exploring the Concept of the Ethical Will as a Way to Leave a Legacy of Values: A Scoping Review

**DOI:** 10.1093/geroni/igab046.2187

**Published:** 2021-12-17

**Authors:** Sarah Neller, Gail Towsley, Mary McFarland

**Affiliations:** 1 University of Utah, Salt Lake City, Utah, United States; 2 University of Utah College of Nursing, Salt Lake City, Utah, United States

## Abstract

Ethical wills communicate a legacy of values through non-legal emotional and supportive instruction to others and are distinct from legal or living wills. Employed for centuries, little is known about how and why ethical wills are used. We conducted the first scoping review on ethical wills to survey the breadth of published information and identify how they are defined and utilized. We followed the Joanna Briggs Institute methodology for scoping reviews employing an a priori protocol and PRISMA-ScR reporting guidelines . We searched 14 databases in November 2019 and January 2021 without filtering publication date or type. Our final extraction form included frequently used terms describing content, purpose, and outcomes. Two reviewers independently screened 1,568 results. Final extraction included 51 documents from 1997-2020, which were primarily published in lay or peer-reviewed journals within law, estate and financial planning, and religion; only 6 research articles were identified. Most frequently, descriptors characterized ethical wills as a non-material legacy of values, beliefs, wisdom, and life lessons learned written to family or future generations. Ethical wills were utilized most to be remembered, address mortality, clarify life’s meaning, and communicate what matters most. They provided opportunity to learn about self, were considered a gift to both writer and recipient and fostered intergenerational interaction and transcendence. Our findings highlight interdisciplinary utilization and dearth of research on ethical wills. Gerontological research is needed to explore ways ethical wills can be used to enhance generativity and intentional living as individuals age and prepare for the end of life.

